# CD155-based chimeric antigen receptor T cells: a promising immunotherapy for cervical and breast cancer

**DOI:** 10.3389/fimmu.2025.1631812

**Published:** 2025-11-19

**Authors:** Jun Ma, Wenjing Zhu, Rui Zhao, Qianqian Shi, Fang Yang, Yangnan Ding, Enwu Yuan, Kai Zhang, Xin Zhao

**Affiliations:** 1 Department of Laboratory Medicine, The Third Affiliated Hospital of Zhengzhou University, Zhengzhou, Henan, China; 2 Tianjian Laboratory of Advanced Biomedical Sciences, Institute of Advanced Biomedical Sciences, Zhengzhou University, Zhengzhou, Henan, China; 3 Zhengzhou Key Laboratory for In Vitro Diagnosis of Hypertensive Disorders of Pregnancy, Zhengzhou, Henan, China; 4 The Radiology Department, The Third Affiliated Hospital of Zhengzhou University, Zhengzhou, Henan, China

**Keywords:** CD155, TIGIT, CAR-T cells, cervical cancer, breast cancer

## Abstract

**Introduction:**

As an immune checkpoint molecule that is overexpressed in cervical and breast cancer, CD155 represents an attractive target for chimeric antigen receptor (CAR) T-cell therapy. However, it is crucial to thoroughly assess the efficacy and safety of CD155-based CAR T cells in preclinical models before considering clinical translation.

**Methods:**

In this study, we developed a CD155-based CAR comprising the extracellular domain of the human TIGIT, 4-1BB, and CD3z signaling domains and utilized a murine model of cervical and breast cancer to comprehensively evaluate the antitumor responses elicited by the CD155-based CAR T cells. The CAR construct was specifically designed to recognize and target CD155-expressing tumor cells.

**Results:**

The results of our study indicated that CD155 exhibits positive staining in the majority of clinical cervical and breast cancer tissues while showing no or low staining in normal tissues. In addition, we observed a correlation between the expression level of CD155 and the proliferation of malignant tumor cells. CD155-based CAR T cells effectively recognize and eliminate CD155-expressing tumor cells *in vitro*. Moreover, *in vivo* experiments using a murine model of cervical and breast cancer revealed that the administration of these CAR T cells leads to significant regression of established tumors without causing any observable toxicity. In addition, the clearance of CD155-positive tumor cells can effectively eliminate tumor cells that exhibit high proliferation rates. This suggests that the treatment approach may offer a safe and effective option for patients with cervical and breast cancer.

**Discussion:**

Overall, our findings provide strong evidence for the efficacy and safety of CD155-based CAR T-cell therapy in cervical and breast cancer. This study contributes to the growing body of research supporting the potential clinical application of CD155-targeted immunotherapy for patients with cervical and breast cancer.

## Introduction

Cervical cancer and breast cancer are two of the most prevalent types of cancer that affect women worldwide (1). These cancers have a significant impact on women’s health and pose a major public health challenge (2). Breast cancer is the most common cancer in women, accounting for a large number of new cases and deaths each year (3). Cervical cancer with higher incidence and mortality rates is observed in low- and middle-income countries that lack organized screening and vaccination programs (4). This highlights the urgent need for the development of innovative therapeutic strategies.

Based on the remarkable success of chimeric antigen receptor (CAR)-modified T-cell immunotherapy in the treatment of hematologic malignancies, researchers have been exploring the application of CAR T-cell therapy in breast and cervical cancer, two prevalent types of cancer affecting women (5, 6). The heterogeneity of breast and cervical cancer poses a significant challenge in their treatment (7). Different subtypes of these two cancers exhibit distinct molecular characteristics and response rates to conventional therapies (8). Currently, numerous clinical trials are underway to assess the effectiveness of CAR T-cell therapy in patients with breast and cervical cancer (9, 10). However, in terms of targeting tumor antigens to treat cervical and breast cancer, the development of effective CAR T-cell therapies has been challenging, in particular the identification of tumor antigens that are highly expressed compared with non-tumor cells and compared with cells in other body organs.

CD155, also called poliovirus receptor (PVR), is a protein that is significantly overexpressed in solid human cancers such as gastric cancer, esophageal cancer, and colorectal cancer, but with limited or no expression in normal tissues (11). An aberrant expression of CD155 on cancer cells is correlated with metastatic potency and poor prognosis (12). As proven in the study by Adhikari et al., brain metastasis-associated fibroblasts secrete CD155 that induces breast cancer invasion (13). In patients with triple-negative breast cancer (TNBC), the expression of CD155 was significantly higher in those with TNM stage II/III/IV disease compared with those with stage I disease. Similarly, the study by Boissiere-Michot et al. reported that a high PVR expression was more frequently observed in tumors with a high histological grade (14, 15). Research by Wu et al. on cervical cancer also demonstrated a gradual increase in CD155 expression with the severity of cervical lesions (16). Together, these findings indicate that the expression of CD155 in both cervical and breast cancer is associated with disease progression. The consistent results across independent cohorts strongly support CD155 as a promising therapeutic target. The CD155/TIGIT axis has attracted considerable interest as an emerging immune checkpoint with potential applications in cancer immunotherapy (17). At present, the main clinical research direction includes recombinant poliovirus-mediated oncolysis therapy and immune checkpoint blocking therapies using anti-CD155 and anti-TIGIT antibodies (18). As an immunoregulatory factor, the blocking of the TIGIT/CD155 pathway improves CD8^+^ T-cell effector function and slows tumor progression (19). However, multiple research groups have discovered the overexpression of TIGIT in natural killer (NK) cells and T cells within peripheral blood mononuclear cells (PBMCs) that exhibit an exhausted phenotype in cervical and breast cancer (20, 21). There have been no reports on the targeting of CD155 for CAR T-cell therapy in cervical and breast cancer.

Safety has been a significant concern in CAR T-cell therapy (22). The limitation for the clinical application of CAR T-cell therapy is its toxicity, including off-tumor toxicity, cytokine release syndrome (CRS), and neurotoxicity (23). Previous studies have shown that the systemic use of monoclonal antibodies can lead to immune-related adverse events (24). For instance, PD1-CD28 CAR T cells secrete higher levels of immune-activated cytokines, which increases the risk of CRS (25). Ligand- or receptor-based CARs have shown encouraging results in the treatment of multiple forms of cancer. One of the key advantages of ligand- or receptor-based CARs over single-chain fragment variables (scFvs) is their low potential for immunogenicity. As the targeting domain of these CARs is derived directly from a natural human protein element, it poses little risk of generating an immune response from the CAR recipient’s host immune system due to immune tolerance for self-antigens (26). Consequently, in this study, the extracellular domain of TIGIT—the natural ligand of CD155—was selected as the recognition moiety of the CAR construct. This approach was adopted with the aim of enhancing the safety profile of the CD155-targeting CAR T-cell therapy.

In this study, we evaluated the expression of CD155 in breast and cervical cancer and assessed the therapeutic efficacy of CAR T cells targeting CD155 in these cancers. To the best of our knowledge, this is the first time that CD155 has been identified as an immunotherapeutic target on patient-derived breast and cervical cancer samples. This work presents a comprehensive investigation into the effectiveness of CD155 CAR T cells using our established mouse model derived from patient-derived breast and cervical cancer cells. These findings highlight the potential of CD155-directed CAR T therapy as a promising approach for improving the prognosis of patients with breast and cervical cancer who currently face a bleak outlook.

## Materials and methods

### Human tissue samples and immunohistochemistry and H-scores

Paraffin-embedded tumor tissues were obtained from The Third Affiliated Hospital of Zhengzhou University. The specimens were collected under an Institutional Review Board (IRB)-approved protocol, with informed consent obtained. Antigen retrieval was performed using the heat-induced epitope retrieval (HIER) method with a microwave oven. The slides were heated in sodium citrate buffer for 15 min at 95°C using a microwave oven, followed by a 30-min cooling period at room temperature. Manual immunohistochemistry (IHC) was optimized and performed using a 1:200 dilution of the antibody against CD155 (no. D8A5G) and Ki67 (no. 9449; both from CST, Danvers, MA, USA). The tissue sections underwent deparaffinization in xylene, dehydration and rehydration in ethanol, and steamer antigen retrieval. Subsequently, the sections were blocked with 10% serum and incubated overnight with primary antibodies at 4°C in a light-protected environment. Afterward, the sections were incubated with the corresponding secondary antibody for 1 h at room temperature. Following chromogenic staining and nuclear counterstaining, the sections were observed under a microscope and photographed (27).

### H&E staining

Tissues from the indicated mouse organs were dissected, fixed in 4% paraformaldehyde for 24 h, embedded in paraffin, and sectioned (4 μm). Paraffin sections were deparaffinized and rehydrated, followed by standard hematoxylin and eosin (H&E) staining (28).

### Cell and culture conditions

The cervical cancer cell lines (HCA1 and CAC1C), the breast cancer cell lines (MDA-MB231 and MCF-7), the immortalized cervical epithelial cell line H8, and the human embryonic kidney cell line 293T were purchased from the Cell Bank of Shanghai Academy of Chinese Sciences (Shanghai, China). All of these cell lines, except for the 293T cell line, were cultured in RPMI 1640 medium (no. 11875093; Gibco, Grand Island, NY, USA) with 5% fetal bovine serum (no. 10099; Gibco) and 100 U/ml penicillin/streptomycin (no. 15140148; Invitrogen, Carlsbad, CA, USA). The 293T cell line was cultured in high-glucose Dulbecco’s modified Eagle’s medium (DMEM) (no. 12491015; Gibco) with 10% fetal bovine serum (no. 10099; Gibco) and 100 U/ml penicillin/streptomycin (no. 15140148; Invitrogen).

### CD155 shRNA and overexpression vector transfection

The CD155 short hairpin RNA (shRNA) lentivirus (shCD155) and the control shRNA lentivirus (NC) were constructed by Gene Pharma (Shanghai, China). The shRNA sequence for shCD155 was 5′-GGGCCAAGTGCACATCATT-3′, while that for NC was 5′-TTCTCCGAACGTGTCACGT-3′. For stable knockdown, the shRNA or the NC was cloned into the pCDH–CMV–shRNA–GFP vector. HCA1 and MDA-MB231 cells were transfected with the NC or the shCD155 lentivirus. The plasmid for overexpressing CD155 was purchased from GenePharma (Shanghai, China). The lentiviral vector pCDH–CMV–CD155–GFP was used for transfection. MCF-7 and CAC1C cells were transfected with the NC or the CD155 lentivirus. To produce lentiviral vectors, the 293T cells were transfected with the shRNA transfer plasmids and packaging plasmids (psPAX2 and PMD2.G) using calcium phosphate precipitation. The viral supernatant was collected at 72 h post-transfection, concentrated via ultracentrifugation, and resuspended for storage at −80 °C. The viral titer [in transducing units (TU) per milliliter] was determined by transducing Jurkat cells with serial dilutions of the concentrate and quantifying the green fluorescent protein (GFP)-positive cells by flow cytometry after 72 h.

### Cell proliferation assays

Colony formation assays were performed in a six-well format. A titration of cell number of each of the cell lines was performed to identify the best cell seeding condition. By day 14, the medium was removed and the cells washed with phosphate-buffered saline (PBS) and stained using 500 μl of 0.005% (*w*/*v*) crystal violet solution in 25% (*v*/*v*) methanol for at least 1 h at room temperature (29). Cell viability was examined using a CCK-8 assay (Sevenbio, Nanjing, China). The cells were seeded in 96-well plates at a density of 4 × 10^3^ cells per well in 200 μl medium for 24, 48, and 72 h. The absorbance was detected at 450 nm after the cells were treated with 10% CCK-8 at 37°C for 2 h. Cell viability was calculated as the ratio of the optical density (OD) values of the drug-treated samples to those of the controls (30).

### CAR design and generation of CAR T cells

The CD155–BBz CAR construct was generated by fusing the extracellular domain of TIGIT derived from the TIGIT cDNA with the transmembrane and cytoplasmic domains of 4-1BB and CD3z. The control CAR (MOCK-CAR) was generated by deleting the BBz.CD3z signaling domain from the CD155-BBz CAR. The CAR sequence targeting CD19, an antigen-irrelevant species-specific negative control, was created using a scFv domain (derived from the FMC63 antibody) and the transmembrane and cytoplasmic domains of 4-1BB and CD3z. All sequences were synthesized by Shanghai Sangon Biotech (Shanghai, China). Human T cells were isolated from the PBMCs obtained from healthy human donors and cervical and breast cancer patients using a human T-cell isolation kit (Miltenyi, Bergisch Gladbach, Germany). Subsequently, the isolated T cells were stimulated with anti-human CD3/28 magnetic beads (no. 11161D; Gibco) for 24 h. The human T cells were cultured in RPMI 1640 medium (no. 11875093; Gibco) supplemented with 5% fetal bovine serum (no. 10099; Gibco) and 100 U/ml penicillin/streptomycin (no. 15140148; Invitrogen). Lentiviruses were produced by transfecting 293T cells using Lipofectamine 3000 (no. L3000001; Invitrogen) according to the manufacturer’s instructions. On day 3, the activated T cells were transduced with the CD155-BBz CAR and CD19-CAR lentiviruses. The transduction efficiency was assessed by measuring the GFP expression using flow cytometry at 7 days post-transduction (31).

### Flow cytometry analysis

For the flow cytometry analysis, the cells were first gated on the forward scatter (FSC) *vs*. the side scatter (SSC) plot to identify the live cell population. Doublets were excluded by gating on FSC-H *vs*. FSC-A. The target positive population was then defined based on the fluorescence intensity using the unstained or isotype control sample as a reference. The cells were stained with ~2 µl antibodies for 30 min at 4°C, washed with PBS (no. P2272; Sigma, St. Louis, MO, USA), and acquired on a flow cytometer (cytoFLEX; Beckman, Brea, CA, USA). PE-anti-human CD155 (no. 337609; BioLegend, San Diego, CA, USA), PE-anti-Ki67 (no. 571599; BD, Franklin Lakes, NJ, USA), PerCP-anti-human CD4 (no. 300529; BioLegend), APC-anti-human CD8 (no. 344746; BioLegend). Analysis was conducted using FlowJo software (FlowJo, Ashland, OR, USA). For intracellular staining, the cells were fixed and permeabilized with Intracellular Fixation and Permeabilization Buffer Set prior to the addition of the intracellular staining antibody sets.

### T-cell functional assays

The cytotoxicity of the CAR T cells was assessed using the lactic dehydrogenase (LDH) release assay (no. ab65393; Abcam, Cambridge, UK) according to the manufacturer’s instructions. The cells were plated in 96-well plates at various effector-to-target (E/T) ratios (i.e., 1:1, 5:1, and 20:1) for a duration of 8 h. The levels of LDH release in the experimental and maximum conditions were measured, and the percentage of cytotoxicity was determined by dividing the experimental LDH release by the maximum LDH release (32). The culture supernatants were collected after 24 h of co-culture to measure the release of interleukin 2 (IL-2) and interferon gamma (IFN-γ) with ELISA following the manufacturer’s instructions (no. S2050 and no. SIF50C; R&D, Minneapolis, MN, USA). After the reactions, the absorbance at 450 nm was read using a spectrophotometer (NanoDrop Technologies, Wilmington, DE, USA). The concentrations of IL-2 and IFN-γ were calculated based on a standard curve (33).

### Animal models

NOD-SCID mice (females, 6–8 weeks old) were obtained from Vital River (Beijing, China) and housed in a specific pathogen-free (SPF) environment. Tumor cells were subcutaneously injected into the hind limb of the mice on day 0. After inoculation, the mice were randomly divided into two groups. CAR T cells, resuspended in 100 µl of PBS, were administered via tail vein injection at the indicated time points. Tumor growth was measured with digital calipers every 2 days. Mice were euthanized when the tumor volume reached 1,500 mm^3^. The tumor volume was calculated using the formula: *V* = (length × width × width) × 0.5. Blood, spleen, and liver samples were collected at the time of sacrifice to determine CAR T-cell infiltration and to assess organ damage severity (34).

### Serum chemistry

For general toxicity due to the CAR T-cell therapy in mice, three groups of non-tumor-bearing mice were included. CD155.CAR, CD19.CAR, or Mock-T cells, resuspended in 100 µl of PBS, were administered via tail vein injection at the indicated time points. The body weight of all mice was measured once every 2 days for 1 month. The serum was collected at the end of the experiment. The activity of serum enzymes such as aldolase, alanine aminotransferase (ALT; Sigma), and creatine kinase (CK) and the serum creatinine values (Abcam, Waltham, MA, USA) were determined following the manufacturer’s protocol (35).

### Statistical analysis

Statistical analyses were performed using GraphPad Prism 8.0 software. Data are displayed as the mean ± SD. Statistical tests included the two-tailed Student’s *t*-test and one-way and two-way ANOVA, with Sidak–Bonferroni correction applied to correct for multiple comparisons, when applicable. Data distribution was assumed to be normal, but which was not formally tested. For *in vivo* experiments, overall survival was depicted by a Kaplan–Meier curve, and a log-rank test was used to compare differences in the survival between groups. ns, *p < 0.05, **p < 0.01, and ***p < 0.001 were considered for statistical significance.

## Results

### CD155 is determined to be a suitable candidate tumor-associated antigen for cervical and breast cancer

**Figure 1:**
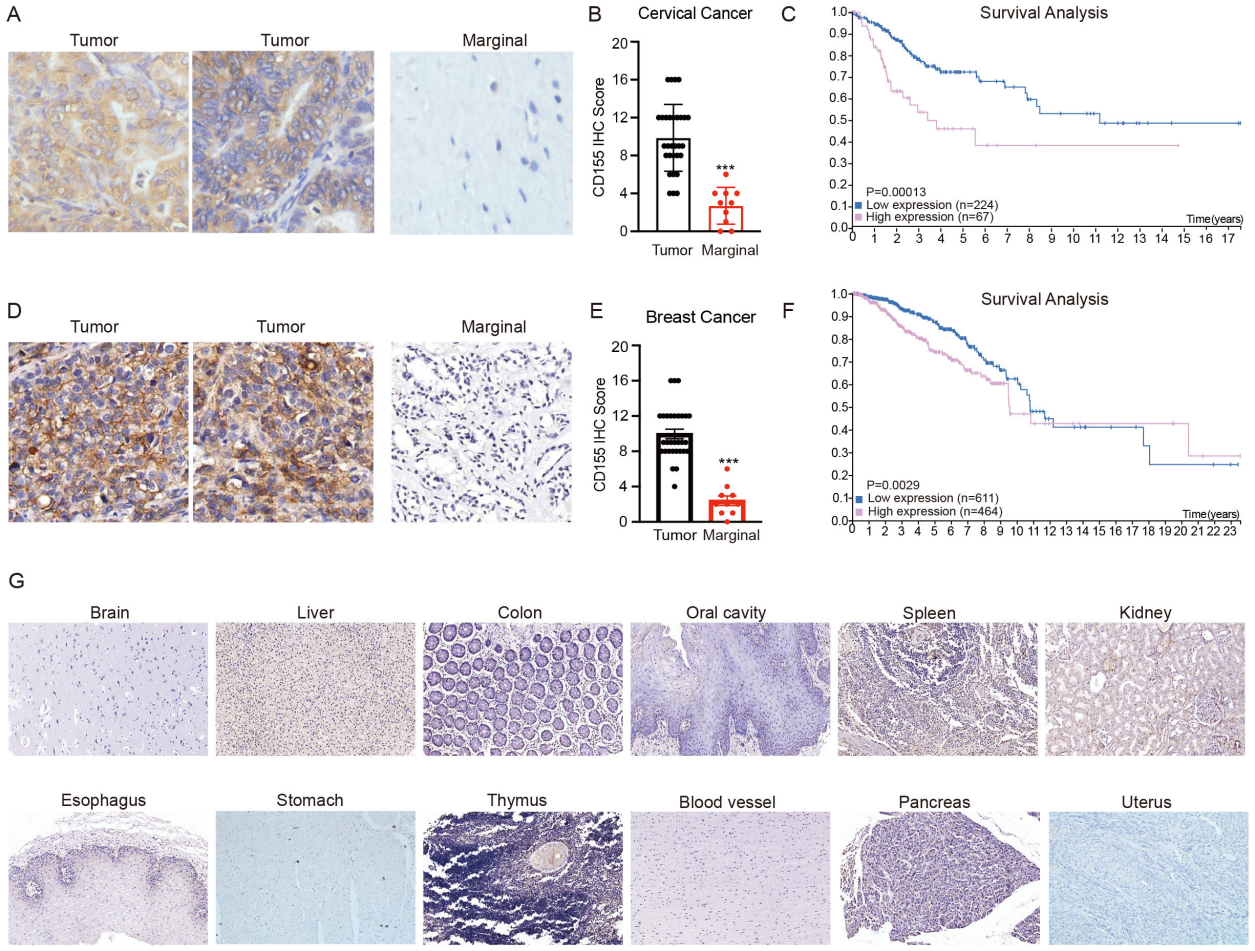
Expression of CD155 in breast cancer, cervical cancer, and normal tissues. **(A)** Representative immunohistochemistry (IHC) of CD155 in cervical cancer and marginal tissues. Note the specific staining in tumor cells and the absence of signal in adjacent stromal tissue, which serves as an internal negative control. **(B)** H-scores were generated by multiplying the percentage of cells positive by the intensity seen for each core. H-scores are shown for cervical cancer (tumor = 30, marginal = 10). **(C)** Survival analysis of cervical cancer patients using The Human Protein Atlas. **(D)** Representative IHC of CD155 in breast cancer and marginal tissues. Note the specific staining in tumor cells and the absence of signal in adjacent stromal tissue, which serves as an internal negative control. **(E)** H-scores were generated by multiplying the percentage of cells positive by the intensity seen for each core. H-scores are shown for breast cancer (tumor = 30, marginal = 30). **(F)** Survival analysis of breast cancer patients using The Human Protein Atlas. **(G)** Representative IHC staining for CD155 expression in normal tissues. ***p < 0.001.

Tumor targeting by CAR T cells requires the expression of tumor-associated antigens (TAAs) on the surface of tumor cells. To investigate the expression of CD155 in primary breast and cervical cancer, we examined the variations in the CD155 expression among breast cancer, cervical cancer, and normal tissues. IHC was conducted using 30 samples of human cervical cancer and 10 samples of peri-cancerous tissues (**Supplementary Table S1**). In addition, 30 samples of human breast cancer paired with adjacent normal mammary tissues were included in the analysis (**Supplementary Table S2**). The results demonstrated that the expression of CD155 in cervical and breast cancer tissues was significantly higher than that in para-carcinomic tissues (**Figures 1A, B, D, E**). To further examine the prognostic potential of CD155 in cervical cancer, we then analyzed the impact of the expression of CD155 on the prognosis of patients with cervical and breast cancer in the Human Protein Atlas database (https://www.proteinatlas.org/). The results showed that the expression of CD155 was negatively correlated with patient survival in cervical and breast cancer (**Figures 1C, F**). Based on the analysis results of IHC and the databases, normal human tissue microarrays were collected and IHC staining performed to examine the expression of CD155. In contrast to malignant samples, the marginal and healthy samples exhibited low levels of CD155 in the limited cells (**Figure 1G**). In general, all of these results provide further evidence of the tumor-specific expression of CD155 at the protein level, suggesting that CD155 could be a potential target for the treatment of cervical and breast cancer.

### CD155 sustains tumor cell proliferation and further promotes tumor progression

**Figure 2:**
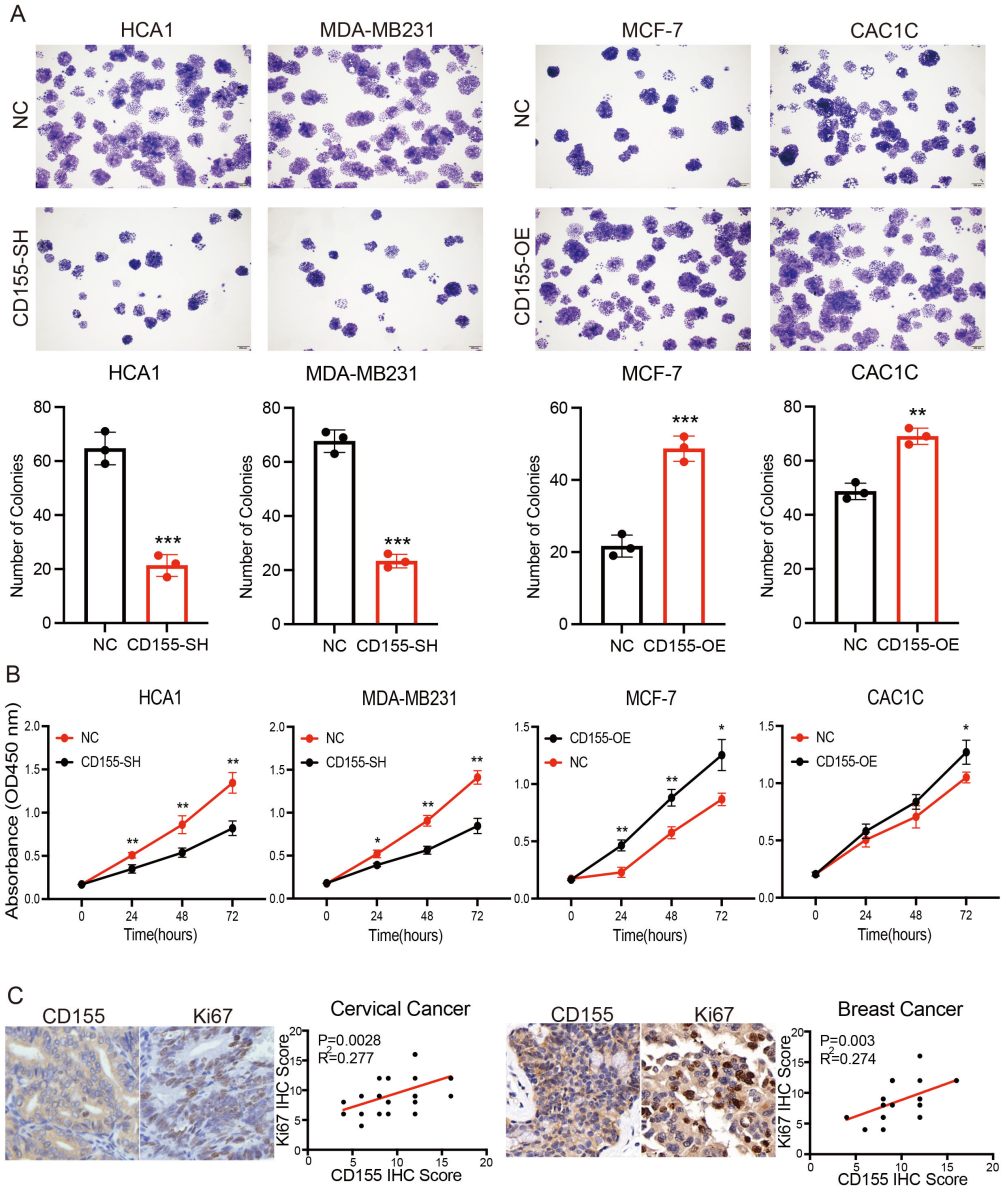
Expression of CD155 promotes breast and cervical cancer progression by increasing tumor cell proliferation. **(A)** Colony formation assay performed in breast and cervical cell lines (*n* = 3). **(B)** CCK-8 assay performed in breast and cervical cell lines (*n* = 3). **(C)** Immunohistochemistry (IHC) analysis showing the correlation between the expression of CD155 and Ki67 in cervical and breast cancer (*n* = 30). *p < 0.05; **p < 0.01; ***p < 0.001.

The available results suggest that CD155 is associated with tumor cell proliferation in the progression of cervical and breast cancer (**Supplementary Figure S1**). To investigate whether CD155 plays a role in the pathogenesis of cervical and breast cancer, we performed gain- and loss-of-function of CD155 in the cervical and breast cancer cell lines. A higher expression of CD155 has been confirmed in two cervical cancer cell lines (HCA1 and CAC1C) and in one breast cancer cell line (MDA-MB231). Conversely, a lower expression of CD155 has been observed in another breast cancer cell line (MCF-7). In addition, the immortalized cervical epithelial cell line (H8) showed no expression of CD155. These findings were confirmed using flow cytometry (**Supplementary Figure S2A**). The overexpression and silencing of CD155 in tumor cell lines were confirmed by flow cytometry (**Supplementary Figure S2B**). The colony formation assays and the CCK-8 assays all showed that CD155 overexpression remarkably promoted, whereas the silencing of CD155 inhibited, the growth of cervical and breast cancer cells (**Figures 2A, B**). We then verified this phenomenon in human tissue specimens. The correlation between CD155 and Ki67 in tumor tissues was examined using IHC, and the results showed that the level of CD155 was positively related to the expression of Ki67 in the cervical and breast cancer tissues (**Figure 2C**). Collectively, our data strongly demonstrated that CD155 serves as a tumor proliferation-promoting factor and may serve to encourage further studies to identify innovative therapeutic approaches for patients with cervical and breast cancer.

### CD155.CAR T cells are cytotoxic to CD155^+^ cervical and breast cancer cell lines *in vitro*

**Figure 3:**
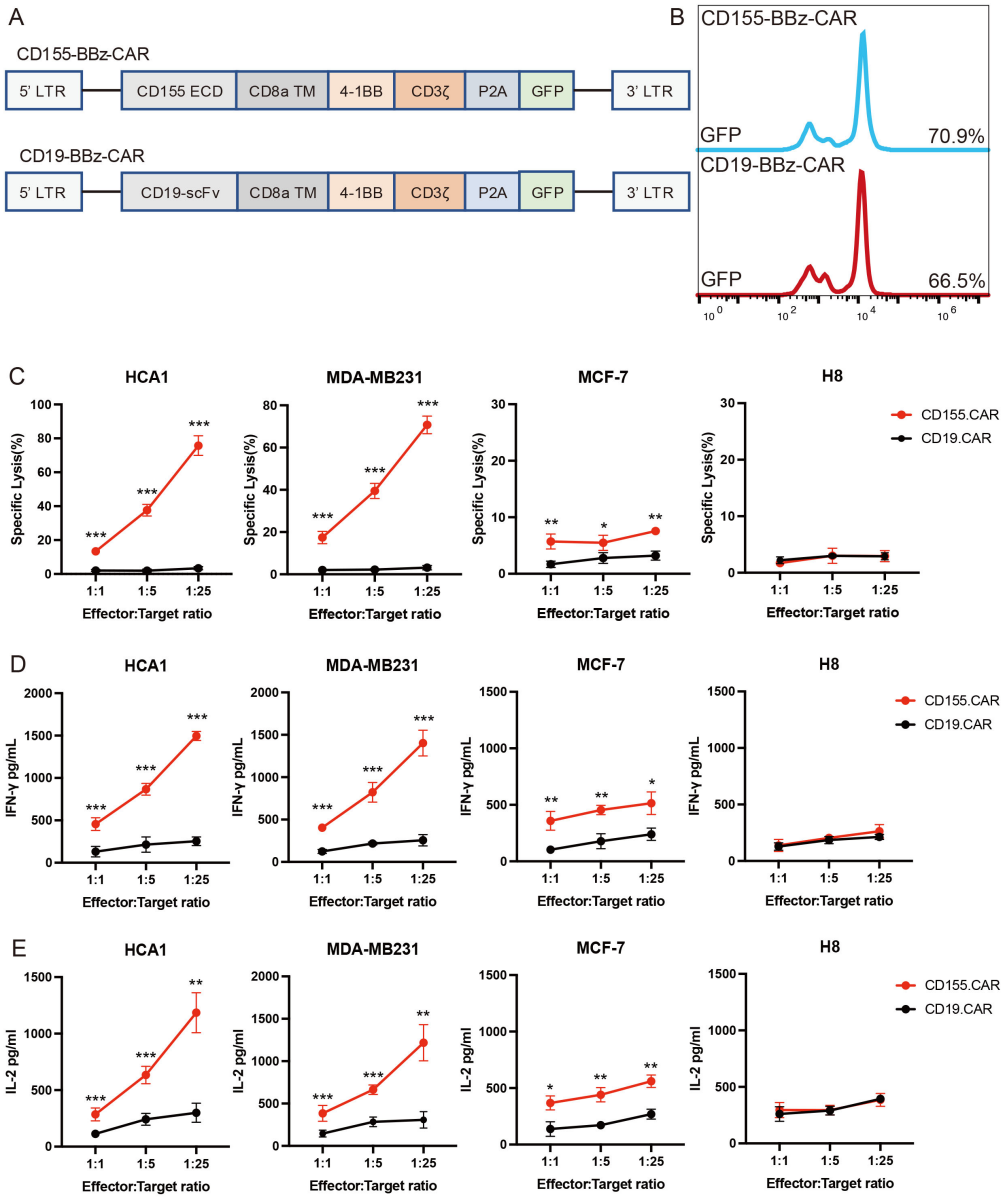
CD155.CAR T cells target CD155^+^ breast and cervical cell lines *in vitro*. **(A)** Retroviral constructs of chimeric antigen receptors (CARs). **(B)** Percentage of green fluorescent protein (GFP) that transduced primary human T cells after 2 weeks detected by flow cytometry. **(C)** Primary T cells from healthy donors transduced with the indicated lentivirus co-incubated with tumor cell lines at varying effector-to-target (E/T) ratios for 12 (h) Cell lysis was determined with the lactate dehydrogenase (LDH) release assay (*n* = 3). **(D)** IFN-γ secretion levels of the T cells co-cultured with target cells for 12 h measured using an ELISA kit (*n* = 3). **(E)** IL-2 secretion levels of the T cells co-cultured with target cells for 12 h measured with an ELISA kit (*n* = 3). *p < 0.05; **p < 0.01; ***p < 0.001.

In general, CAR T-cell therapy is antigen-specific, and its efficacy is dependent on the level of expression of the targeted antigen on the tumor cell (36). To evaluate the CD155 antigen specificity, we transduced the activated T cells either with a second-generation CD155-specific CAR (CD155.CAR) or a CD19 control CAR (CD19.CAR) (**Figure 3A**). The CD155-specific CAR expression on human T cells transduced with lentiviral particles was analyzed using flow cytometry through detection of GFP fluorescence (**Figure 3B**). We then evaluated the function of the CAR T cells against the CD155^+^ tumor lines by assessing the cytolytic potential, the proliferative capacity, and the cytokine secretion. To evaluate the cytolytic potential of the CD155.CAR T cells, we performed a 12-h co-incubation experiment against HCA1, MDA-MB231, MCF-7, and H8. All of the CD155^+^ cervical and breast cancer cell lines tested exhibited cell killing, even at low E/T ratios. However, the CD155 low or negative cell lines did not show sensitivity to killing by CD155.CAR T cells (**Figure 3C**). Consistently, the CD155.CAR T cells upregulated the secretion of IFN-γ and IL-2 compared with the Ctrl-T cells (**Figures 3D, E**). Taken together, these data suggest that CD155.CAR T cells exhibit specificity and cytotoxicity against CD155^+^ cervical and breast cancer cells through greater secretion of IL-2 and IFN-γ.

### Antitumor efficacy of CD155.CAR T cells in xenograft mouse models of breast and cervical cancer

**Figure 4:**
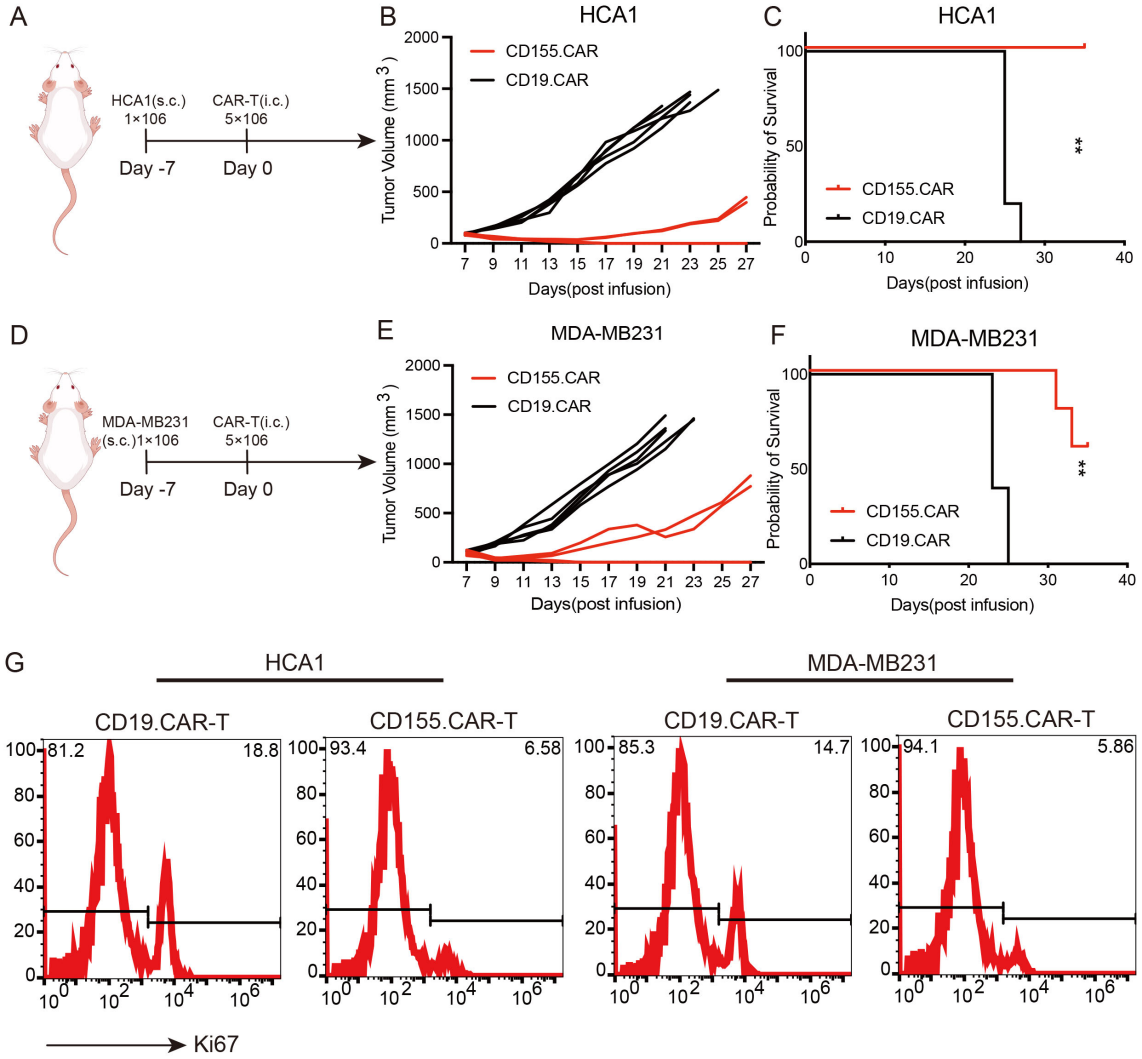
Antitumor efficacy of CD155.CAR T cells *in vivo*. **(A)** Schematic representation of the cervical cancer xenograft model. **(B)** Growth curve of the HCA1 xenografts treated with the indicated T cells (*n* = 5). **(C)** Survival analysis of the mice in the two groups (*n* = 5). **(D)** Schematic representation of the breast cancer xenograft model. **(E)** Growth curve of the MDA-MB231 xenografts treated with the indicated T cells (*n* = 5). **(F)** Survival analysis of the mice in the two groups (*n* = 5). **(G)** Representative flow cytometry images with Ki67. Tumor tissues were harvested 14 days after the indicated T-cell infusion. **p < 0.01.

To evaluate the *in vivo* tumor suppression activity of the CD155.CAR T cells, a cervical cancer xenograft mouse model was established by the subcutaneous transplantation of HCA1 cells in NOD-SCID mice. After 7 days of tumor cell implantation, the mice received CAR T treatment via tail vein injection (**Figure 4A**). Compared with the control group, the CD155.CAR T cells were effective in controlling the growth of CD155^+^ tumors and showed safety profiles as indicated by the body weight loss (**Figures 4B, C**). As a result, the mice in the experimental groups received CD155.CAR T cells, which significantly extended the overall survival (**Figure 4D**). Similar efficacy results were observed in the breast cancer xenograft model established by the subcutaneous transplantation of MDA-MB231 cells in NOD-SCID mice (**Figures 4E–H**). At the end of the treatment study, the remaining tumor xenograft samples were collected to examine the proliferation of tumor cells. As shown in **Figure 4I**, in the CD155.CAR T-cell treatment group, the proliferation of tumor cells was significantly decreased as determined by Ki67 staining. Collectively, these *in vivo* experiments indicated that CD155.CAR T cells improve the control of established cervical and breast cancer xenografts of tumor-expressing CD155 target antigens.

### Safety evaluation of CD155.CAR T cells

**Figure 5:**
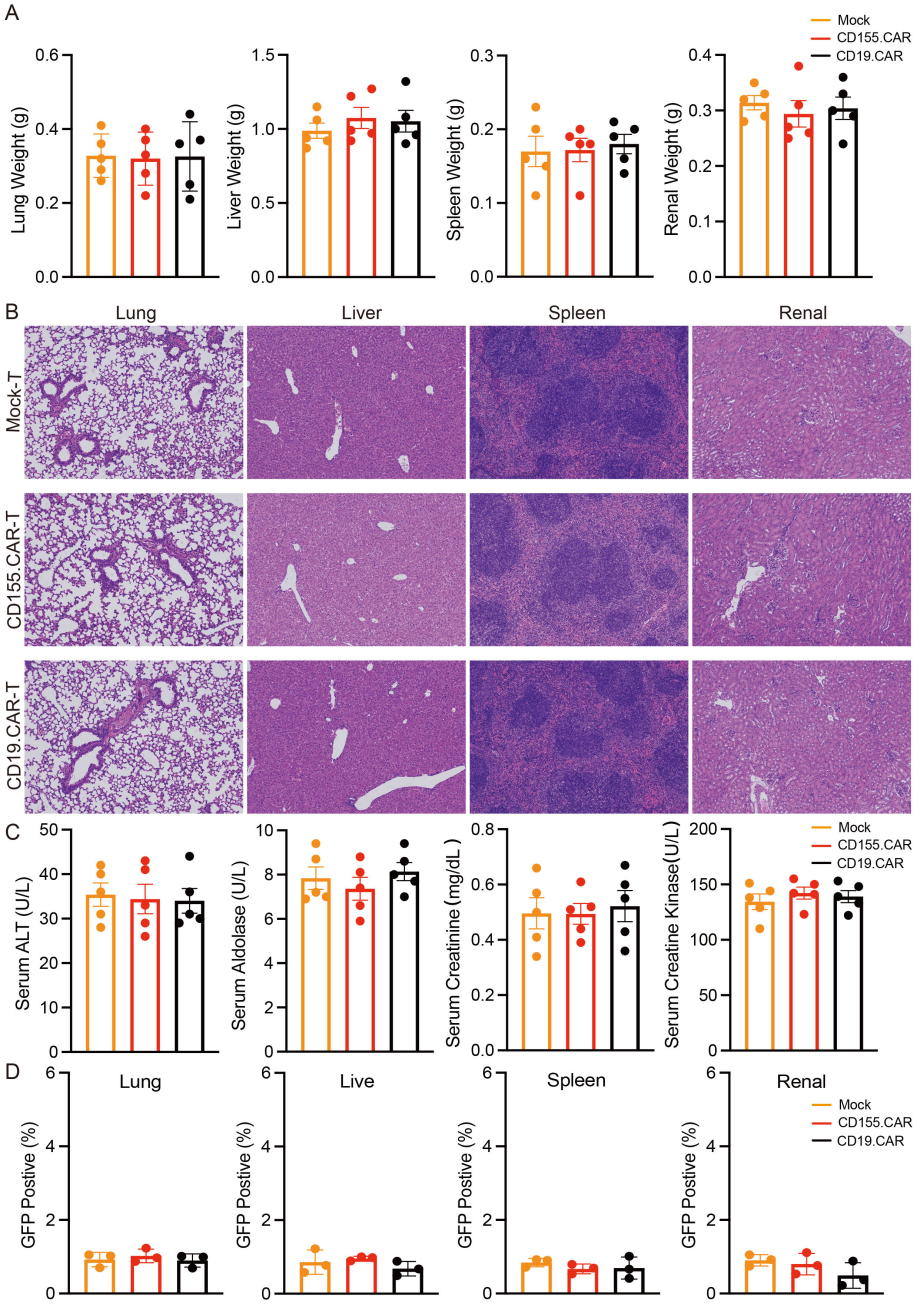
CD155.CAR T cells show a good safety profile in mice. **(A)** Weights of the liver, spleen, lung, and kidneys (*n* = 5). **(B)** H&E staining of the indicated organs. **(C)** Serum chemistry analysis for the activity of serum aldolase, alanine aminotransferase (ALT), and creatine kinase (CK) and for creatinine (*n* = 5). **(D)** Flow cytometry staining of the lung, liver, spleen, and kidneys of mice with green fluorescent protein (GFP) to detect CAR T-cell infiltration.

Although the low or negative expression of CD155 on normal cells was confirmed, as shown in **Figure 1**, it was also necessary to evaluate the safety after CD155.CAR T-cell therapy. The general toxicity in non-tumor-bearing mice due to the CAR T-cell administration was evaluated in non-tumor-bearing NOD-SCID mice as mice were not expected to express high levels of CD155 in any organ. To determine the safety profile of the CD155.CAR T cells and to further discuss their translation into the clinic, the non-tumor-bearing mice in each group received CD155.CAR-T, CD19.CAR-T, or Mock-T cells infused via the tail vein. The mice were monitored for 30 days and were collected to determine the weights of the liver, spleen, lung, and kidneys. There were no significant differences in the weights of these organs, demonstrating the good safety profile of the CD155.CAR T cells (**Figure 5A**). Further histological analysis of the liver, the spleen, the lung, and the kidneys by H&E staining revealed no obvious abnormalities in the CD155.CAR T-cell-treated mice (**Figure 5B**). CAR T-cell administration showed a remarkable safety profile in terms of changes in the serum enzymes such as aldolase, ALT, CK, and serum creatinine (**Figure 5C**). There was no significant decrease in the body weights of the mice in the three groups (**Supplementary Figure S3**). Furthermore, we investigated whether the injected T cells might colonize other organs to cause tissue injury. There was only minimal infiltration of GFP-positive CAR T cells in all of the examined tissues across all treatment groups (**Figure 5D**). This finding provides strong evidence that, in the absence of substantial antigenic stimulation, CD155.CAR T cells do not preferentially expand, persist, or accumulate in these vital organs. Together, CD155.CAR T cells were well tolerated and did not result in obvious adverse events in mice, suggesting that CD155 is a safe and potent antitumor target for CAR T cells and is worthy of further development for clinical translation.

### CD155.CAR T cells derived from patients with cervical and breast cancer showed antitumor activity

**Figure 6:**
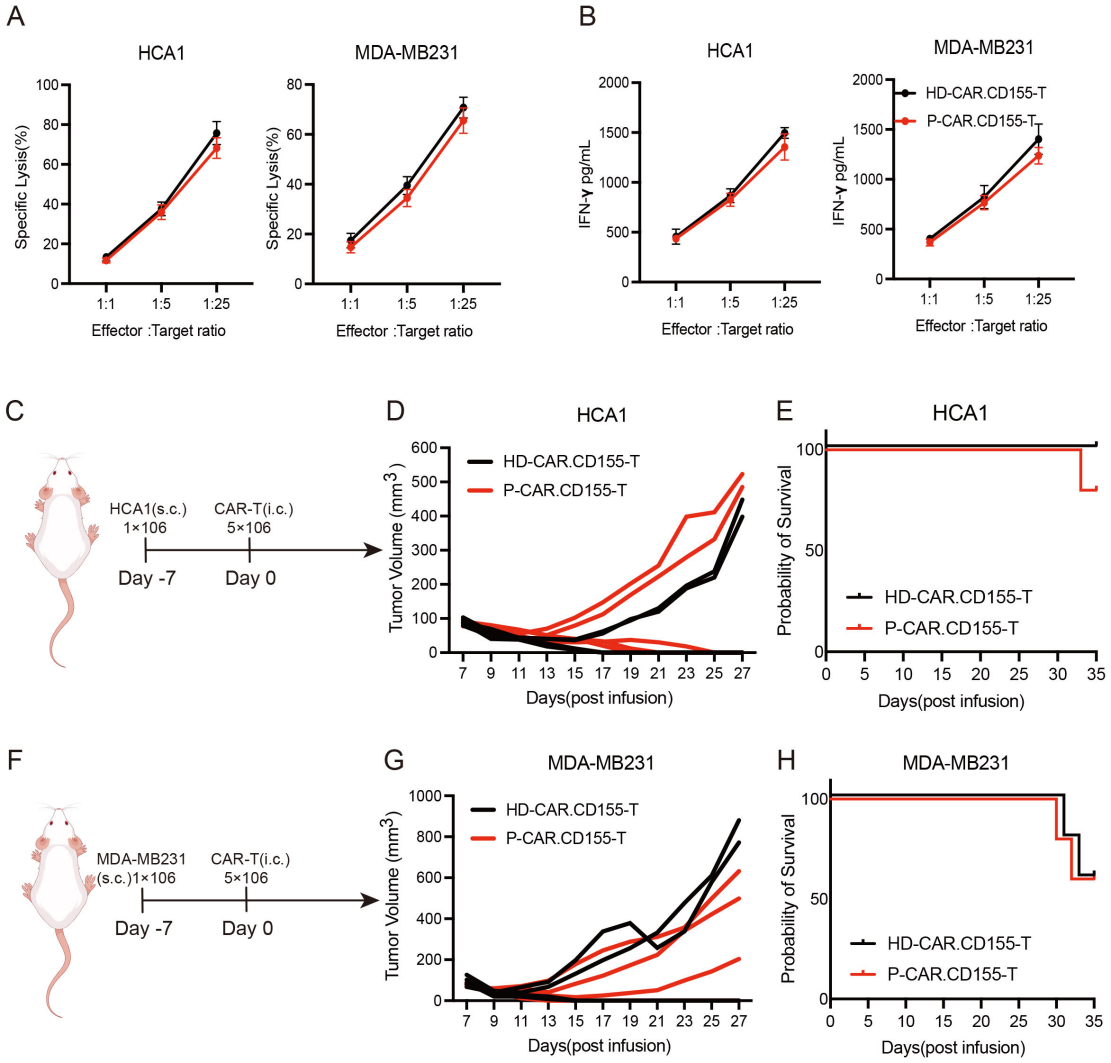
Patient-derived CD155.CAR T cells potently suppress breast and cervical cancer both *in vitro* and *in vivo*. **(A)** Primary T cells from healthy donors or patients transduced with the indicated lentivirus co-incubated with tumor cell lines at varying effector-to-target (E/T) ratios for 12 (h) Cell lysis was determined with the lactate dehydrogenase (LDH) release assay (*n* = 3). **(B)** IFN-γ secretion levels of the T cells co-cultured with target cells for 12 h measured with an ELISA kit (*n* = 3). **(C)** Schematic representation of the cervical cancer xenograft model. **(D)** Growth curve of the HCA1 xenografts treated with the indicated T cells (*each line* represents the tumor growth curve of an individual mouse, *n* = 5). **(E)** Survival analysis of the mice in the two groups (*n* = 5). **(F)** Schematic representation of the breast cancer xenograft model. **(G)** Growth curve of the MDA-MB231 xenografts treated with the indicated T cells (*each line* represents the tumor growth curve of an individual mouse, *n* = 5). **(H)** Survival analysis of the mice in the two groups (*n* = 5).

Patients with cervical and breast cancer have reported abnormalities in their lymphocyte function. Thus, the antitumor activity of the CD155.CAR T cells derived from cervical and breast cancer patients’ T cells needed to be verified. The PBMC collected from one patient with cervical cancer [squamous cell carcinoma, human papillomavirus (HPV)-positive, TNM stage II] was used to isolate the T cells, and the T cells were then used for transduction with the CD155-BBz CAR lentiviruses (**Supplementary Figure S4A**). In agreement with previous reports, the patient’s T cells (P-T cells) proliferated more slowly than the T cells of a healthy donor (HD-T cells) (**Supplementary Figure S4B**). However, the patient’s T cells had similar CD4/CD8 ratios to those of the T cells derived from the healthy donor (**Supplementary Figure S4C**). To determine the cytotoxicity of the CD155.CAR T cells from different sources against the CD155+ tumor cells, a co-incubation experiment that included both T cells and tumor cells was performed. The results showed that the patient’s CD155.CAR T cells had a comparable antitumor activity to the CD155-based CAR T cells derived from the healthy donor (**Figures 6A, B**). Correspondingly, both CD155.CAR T cells showed effective antitumor activity against the xenografts formed by the HCA1 and MDA-MB231 cells in NOD-SCID mice (**Figures 6C–H**).

## Discussion

While the majority of cervical and breast tumors diagnosed at the early stages are currently being successfully treated with surgery combined with adjuvant precision therapies, advanced tumors still pose an unmet medical need (37, 38). Despite extensive exploration in preclinical and clinical trials for cervical and breast tumors, CAR T-cell-based immunotherapy has struggled to replicate the success observed with CD19.CAR T cells in hematopoietic malignancies (5, 39). One major obstacle impeding the potency of CAR T cells in solid tumors is the absence of an ideal surface antigen (40). Here, we demonstrated that CD155 is expressed in cervical and breast tumors and that CD155 effectively promoted tumor growth in the cervical and breast xenograft models. This finding makes CD155 an attractive target for T-cell therapies. We showed that CD155.CAR T cells have potent antitumor activity against cervical and breast tumor cells *in vitro* and *in vivo*.

As an inhibitory marker, CD155 interacts mainly with the T-cell immunoreceptor with the immunoglobulin (Ig) and immunoreceptor tyrosine-based inhibitory motif (ITIM) domains (TIGIT) in cytotoxic cells (41). Multiple studies have reported on the role of CD155 and its receptor in the progression of various tumors, including multiple myeloma, cervical cancer, breast cancer, lung cancer, and immune evasion (20, 41, 42). Sun et al. found that CD155 was an independent risk factor and that the expression of CD155 was significantly positively correlated with tumor, node, and metastasis (43). CD155 has also been reported to be involved in various processes of tumor occurrence and development, including tumor cell proliferation, invasion and migration, and angiogenesis, and to importantly play a pivotal role in tumor immune escape (44, 45). CD155 is arguably the most targeted receptor in the development of cancer therapies as it is highly expressed across multiple tumor types, but exhibits restricted expression in normal tissues (46). In our IHC results, the expression of CD155 was absent or weak in normal tissues, but could be detected at high expression levels in cervical and breast tumor cells. The survival analysis using the Human Protein Atlas further confirmed the significant negative association of CD155 with patients’ death. These results showed the potential of CD155 as a therapeutic target in immunotherapy for patients with breast or cervical tumor.

Uncontrolled cell proliferation is one of the most important characteristic features of cancer, including cervical and breast cancer (47). A low-frequency Ki67^+^ subclone may initiate the tumor and then persist throughout treatment by generating a cellular hierarchy, with cells endowed with a Ki67-driven self-renewal phenotype being able to escape such therapies and reemerge to initiate tumor relapse (48). Ki67 upregulation may help drive the proliferation of malignant tumor cells, making it a potential biomarker of tumor aggressiveness and poor prognosis (49). Thus, in the context of cancer treatment, the elimination of highly proliferative tumor cells has been shown to effectively control the progression of tumors. Here, we have provided evidence that the CD155 protein expression in breast and cervical cancer showed a statistically significant positive correlation with Ki67 expression. Thus, specifically blocking CD155 expression in human tumor cells decreased the primary tumor growth in xenograft models, which is consistent with a previous study by Zhang et al. (16, 50). Based on these results, the elimination of targeted CD155^+^ tumor cells by CAR T cells was substantially more efficient than that by others. However, it should be noted that this needs to be further evaluated in extensive clinical trials.

CD155 interacts with several CD155-like proteins, including TIGIT, CD96, DNAM-1, and CD112R. Within this signaling network, the interaction between CD155 and TIGIT plays a particularly important role in immune inhibition. In addition, CAR T-cell dysfunction associated with TIGIT expression contributed to poor responses in patients with relapsed or refractory non-Hodgkin’s lymphoma. CD155 binds to the receptors TIGIT, CD96, and CD226 with different affinities, thus exerting an immunomodulatory effect. Among them, CD155 had the highest affinity with TIGIT (18). To identify the binding partners of TIGIT, Xin et al. screened a large library of secreted proteins with the ForteBIO Octet system and found that TIGIT effectively blocked the interaction of CD155 with its other receptors (51). We leveraged this property in our study. Specifically, we employed the extracellular domain of human TIGIT as a recognition sequence to attenuate a negative checkpoint in exhausted T and NK cells. This advantage allows patients to better harness the synergy of their own immune system while undergoing CAR T-cell therapy. CD155-targeted therapy has been widely considered, with the study by Wu et al. representing the first time that CD155 has been used as a target for CAR T-cell treatment of melanoma (52). In their study, the extracellular region of DNAM-1 was chosen as the recognizing domain for CAR T cells to target CD155. The study by Pan et al. selected multiple sequences as recognizing domains for CAR T cells combined with NK-92 cells to target CD155 in glioblastoma (53). In their study, CD155 targeting CAR T cells exhibited a killing capacity against glioma stem cells. All these results confirmed the feasibility of CD155 as a potential target for tumor therapy. In our previous studies, CD155 was a promising target for digestive system cancer therapy, inducing complete tumor regression and long-lasting immunological memory of established solid tumors in xenograft models. We further explored the ability of CD155-directed CAR T cells for specific depletion of the immunosuppressive subset of tumor-associated macrophages (TAMs) (54). In this study, CD155 was first proposed for CAR T-cell therapy in breast and cervical cancer. Our results not only confirmed the efficacy of CD155-directed CAR T cells in breast and cervical cancer but also further confirmed the safety of these CAR T cells by H&E staining. As shown in our H&E staining studies, significant tissue injury was not observed in the multiple organs examined after intravenous injection of CAR T cells. However, the biology, physiology, and immunology of mice are quite different from those of humans. To confirm the safety of these CAR T cells more accurately, further work such as a safety evaluation in non-human primates will be necessary.

A growing body of literature has confirmed that CD155 is expressed in a significant proportion of estrogen receptor- and progesterone receptor-positive (ER+/PR+) breast cancer patient tissues. For instance, a recent study specifically investigated the expression of CD155 across molecular subtypes in a cohort of patients with breast cancer undergoing neoadjuvant chemotherapy (NACT) (14). This study confirmed that while CD155 is most highly expressed in TNBC, it is also readily detectable in the luminal (ER+/PR+) and HER2+ subtypes. Furthermore, a high CD155 expression is consistently associated with poor prognostic factors across breast cancer subtypes, such as lymph node metastasis and reduced survival. Therefore, our choice of CD155 as a target is strongly supported by its clinical relevance in a broad spectrum of breast cancers, including the ER+/PR+ subset. The literature unequivocally demonstrates that conventional therapies, especially chemotherapy, can modulate the expression of CD55. Crucially, a persistently high CD155 expression after NACT was significantly correlated with poor treatment response and worse survival outcomes, particularly in patients with advanced lymph node involvement. This identifies CD155 as a potential mediator of therapy resistance. Tumors may upregulate immunomodulatory molecules such as CD155 to evade immune surveillance following chemotherapy-induced stress. This suggests that CD155-targeted therapies, such as our CAR T cells, are ideally suited as a subsequent-line treatment to eliminate residual, therapy-resistant CD155-high cancer cells that remain after conventional therapy. This positions our approach not as a replacement for but as a powerful adjuvant to existing standard-of-care treatments. The fact that its expression is modulated by and is associated with resistance to conventional therapy strengthens, rather than weakens, the rationale for our CD155-directed CAR T-cell strategy.

In conclusion, our results indicated that CD155 is expressed in breast and cervical cancer and that its expression level is correlated with the proliferative ability of these cancers and the poor survival of patients with these malignancies. The overexpression of CD155 was also observed in both primary breast and cervical cancer cells and in breast and cervical cancer cell lines. CD155-specific CAR T cells presented obvious cytotoxic effects on breast and cervical cancer cells *in vitro* and significantly induced tumor regression in orthotropic breast and cervical cancer models. Our study highlights the use of CD155-directed CAR T cells as a promising therapeutic strategy for solid tumors. However, this study has several limitations. The regulation of the tumor microenvironment requires further verification. Trafficking CAR T cells into malignant tissues is critical for tumor eradication. In addition, there is a lack of a clinically relevant tumor model, such as patient-derived xenograft (PDX) and humanized mouse models, that would be invaluable for further investigation of the potential of CD155-targeted CAR T-cell therapy to regulate the endogenous immune microenvironment.

## Data Availability

The raw data supporting the conclusions of this article will be made available by the authors, without undue reservation.
