# Supplementary material for: CD155-based chimeric antigen receptor T cells: a promising immunotherapy for cervical and breast cancer
Source: Front Immunol. 2025 Nov 19;16:1631812. doi: 10.3389/fimmu.2025.1631812 (PMC12696411; doi:10.3389/fimmu.2025.1631812)
Supplement: Supplementary file 1 [file DataSheet1.docx]

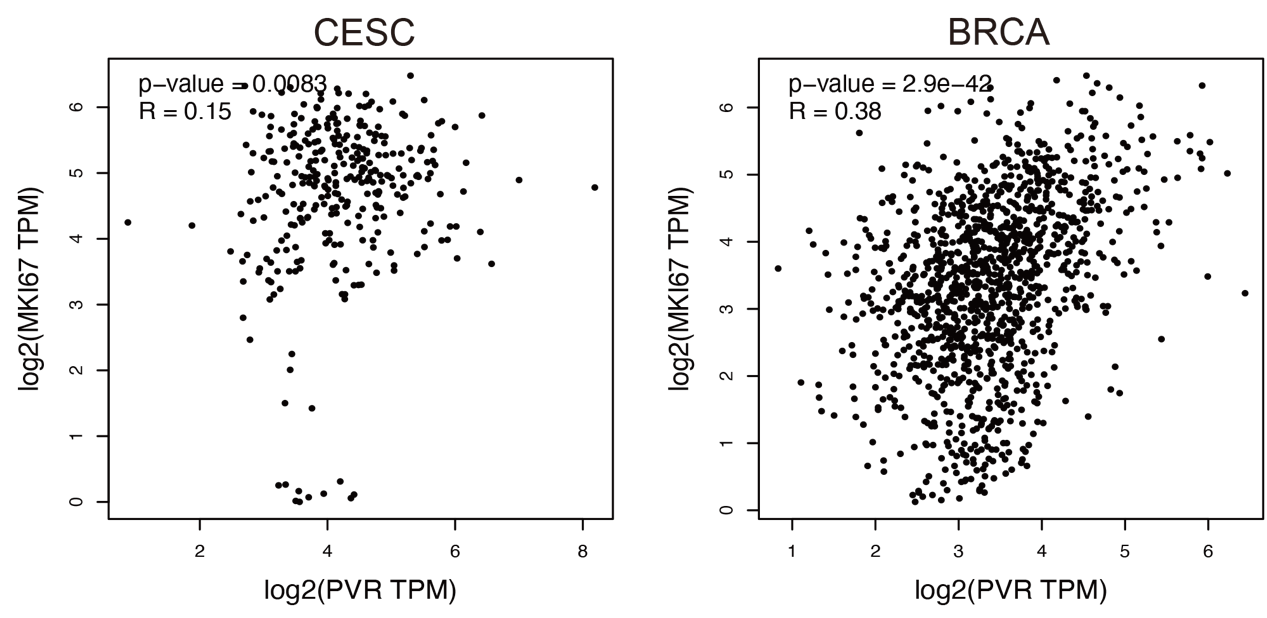


**Figure S1. TCGA data indicated that CD155 expression showed a significant positive correlation with MKi67 in** **breast and cervical cancers.**


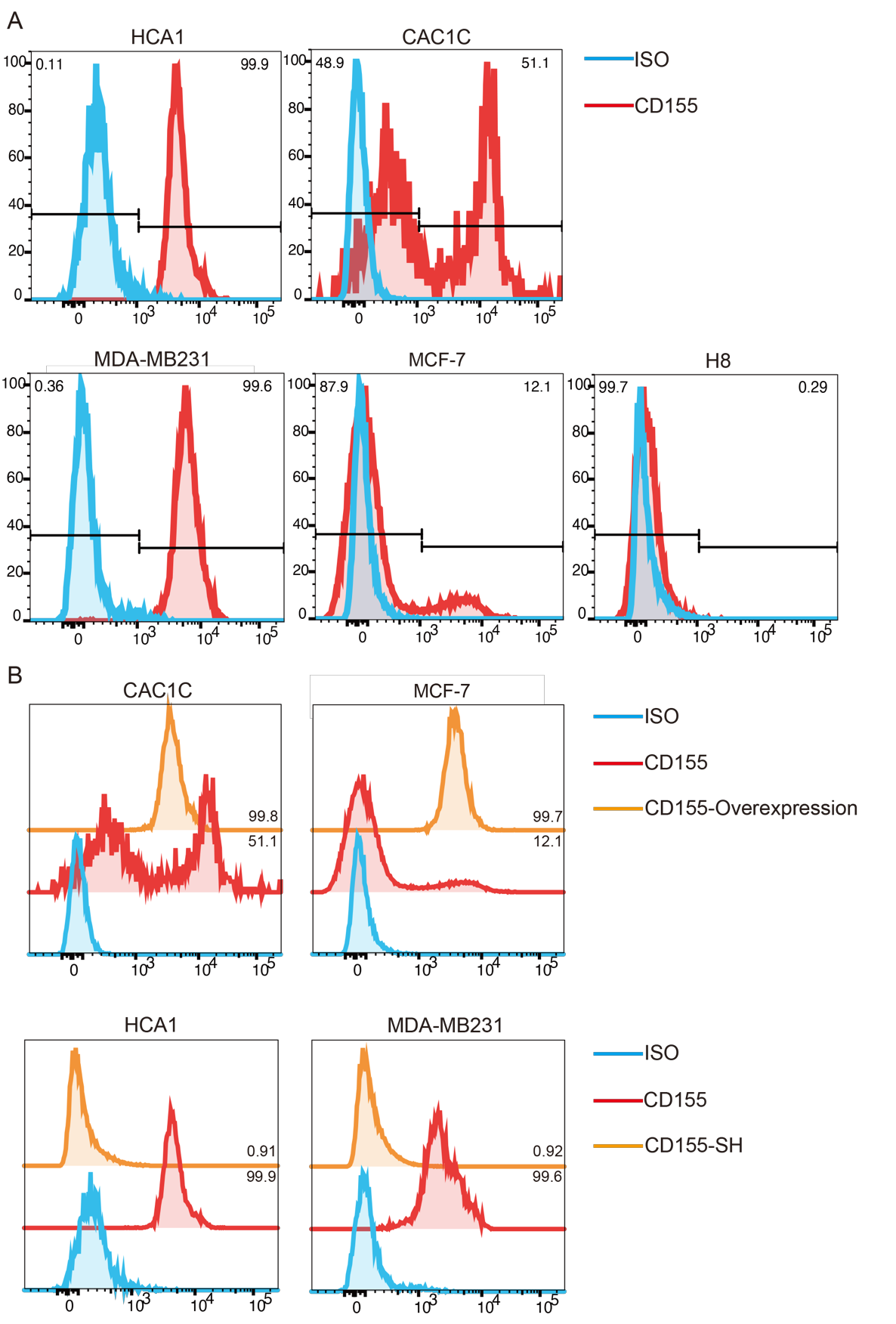


**Figure S2. The expression of CD155 in cancer cell lines.** (A) The expression of CD155 in breast and cervical cell lines was detected by flow cytometry. (B) Flow cytometry analysis of CD155 expression levels in breast and cervical cell lines after overexpression or SH.


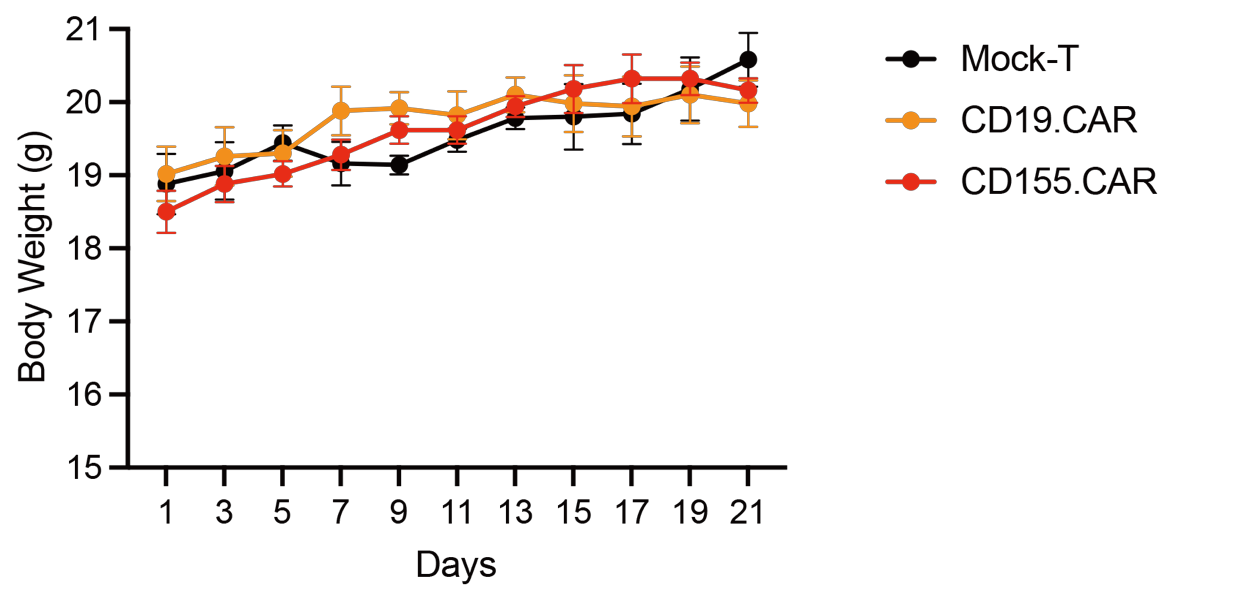


**Figure S3. CD155.CAR-T cells did not decrease the body weight in mouse tumor model.**


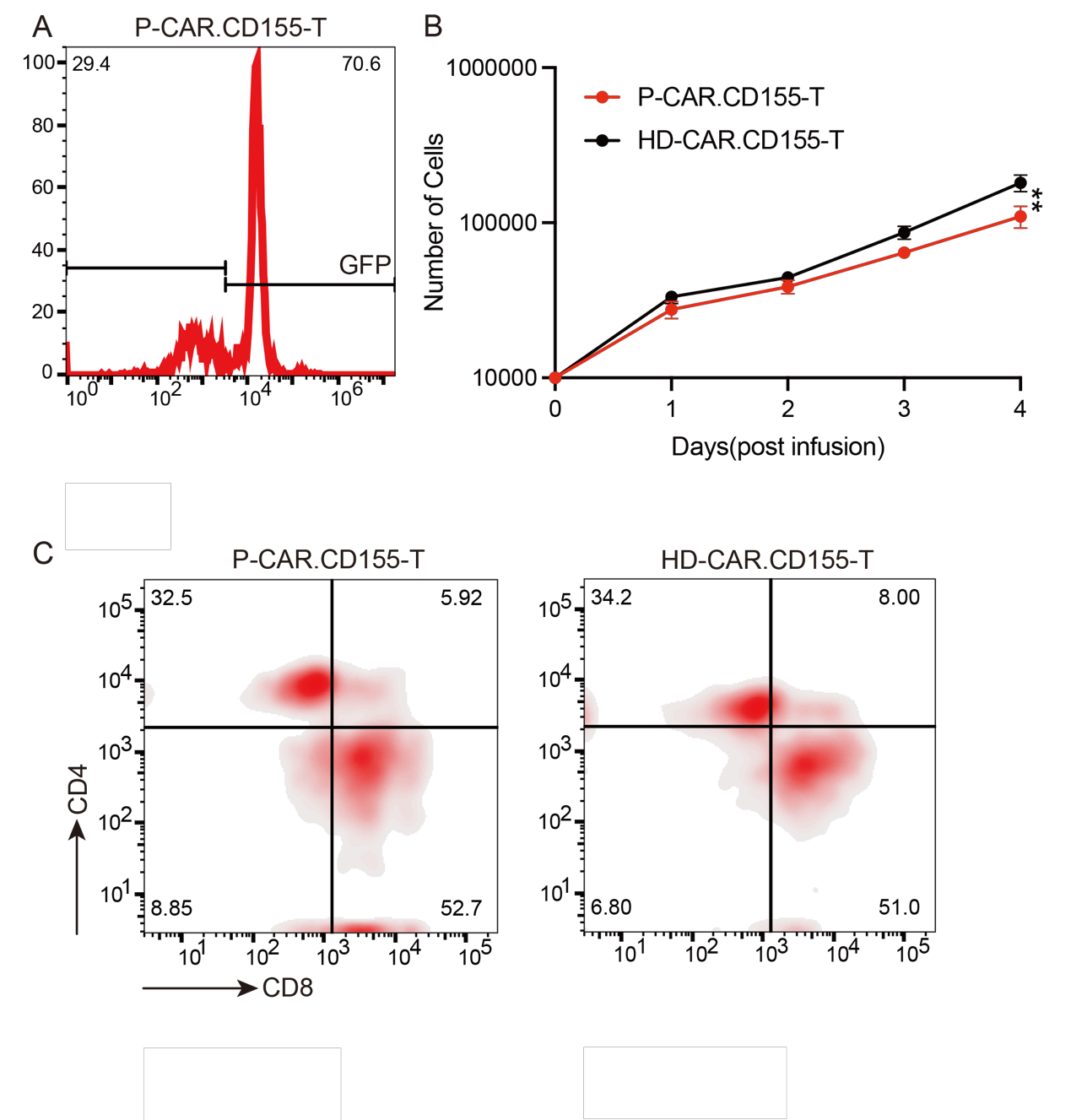


**Figure S4. CAR-T cells from patient followed a pattern comparable to that from healthy donor.** (A) Flow cytometry analysis of the expression of CD155.CAR in transduced T cells isolated from patient. (B) Growth curves of CAR-T cells isolated from healthy donor and patient. (C) Proportions of CD4+ and CD8+ T cells in CD155.CAR -T cells from healthy donor and patient by flow cytometry. ***P* < 0.01

Table S1. Clinical information of Cervical cancer patients.

| Factors | Total |
| --- | --- |
| Age (y) |  |
| ≤ 50 | 16 |
| > 50 | 14 |
| TNM stage  II  III  IV  HPV  Negative  Positive  Histopathology  Adenocarcinoma  Squamous cell carcinoma | 8  10  12  23  7  7  23 |

Table S2. Clinical information of breast cancer patients.

| Factors | Total |
| --- | --- |
| Age (y) |  |
| ≤ 50 | 19 |
| > 50 | 11 |
| TNM stage  II  III  IV  ER  Negative  Positive  PR | 6  9  15  16  14 |
| Negative  Positive  HER2  Negative  Positive  TNBC | 17  13  20  10  4 |
